# A novel vascular endothelial growth factor-directed therapy that selectively activates cytotoxic prodrugs

**DOI:** 10.1038/sj.bjc.6600911

**Published:** 2003-05-13

**Authors:** R A Spooner, F Friedlos, K Maycroft, S M Stribbling, J Roussel, J Brueggen, B Stolz, T O'Reilly, J Wood, A Matter, R Marais, C J Springer

**Affiliations:** 1 Cancer Research UK Centre for Cancer Therapeutics at the Institute of Cancer Research, 15 Cotswold Road, Sutton, Surrey SM2 5NG, UK; 2 Cancer Research UK Centre for Cell and Molecular Biology at the Institute of Cancer Research, 237 Fulham Road, London SW3 6JB, UK; 3 Novartis Pharmaceutical AG, Pharmaceutical Research, CH-4002, Basel, Switzerland

**Keywords:** CPG2, VEGF, fusion proteins, targeting

## Abstract

We have generated fusion proteins between vascular endothelial growth factor (VEGF) and the bacterial enzyme carboxypeptidase G2 (CPG2) that can activate the prodrug 4-[(2-chloroethyl)(2-mesyloxyethyl)amino]benzoyl-L-glutamic acid (CMDA). Three asparagine residues of CPG2 were mutated to glutamine (CPG2(Q)3) to prevent glycosylation during secretion, and truncations of VEGF_165_ were fused to either the C- or N-terminal of CPG2. The *K*_m_ of the fusion proteins (37.5 *μ*M) was similar to that of secreted CPG2(Q)3 (29.5 *μ*M) but greater than that of wild-type CPG2 (8 *μ*M). The affinity of the fusion proteins for VEGF receptor-2 (VEGFR2) (*K*_d_=0.5–1.1 nM) was similar to that of [^125^I]VEGF (*K*_d_=0.5 nM) (ELISA) or slightly higher (*K*_d_=1.3–9.6 nM) (competitive RIA). One protein, VEGF_115_-CPG2(Q)3-H_6_, possessed 140% of the enzymic activity of secreted CPG2(Q)3, and had a faster half-maximal binding time for VEGFR2 (77 s), than the other candidates (330 s). *In vitro*, VEGF_115_-CPG2(Q)3-H_6_ targeted CMDA cytotoxicity only towards VEGFR-expressing cells. The plasma half-life of VEGF_115_-CPG2(Q)3-H_6_ *in vivo* was 3 h, comparable to equivalent values observed in ADEPT. We conclude that enzyme prodrug therapy using VEGF as a targeting moiety represents a promising novel antitumour therapy, with VEGF_115_-CPG2(Q)3-H_6_ being a lead candidate.

Neoangiogenesis is critical for the growth, malignant progression, and metastatic spread of solid tumours and is effected by the release of angiogenic factors that are secreted by tumour cells (Fidler and Ellis, 1994; Folkman, 1995; Fukumura *et al*, 1998). One such angiogenic factor is vascular endothelial growth factor (VEGF) (Tischer *et al*, 1989). VEGF homodimers act by binding to two cell surface receptors (VEGFRs), known variously as VEGFR-1, *flt-1*, or fms-like tyrosine kinase, and VEGFR2, KDR, kinase insert domain-containing receptor, or *flk-1*. Alternate splicing of the VEGF transcript results in the expression of five isoforms of VEGF, comprising molecules consisting of 208, 189, 165, 145, or 121 amino acids (Leung *et al*, 1989; Tischer *et al*, 1989; Houck *et al*, 1991; Poltorak *et al*, 1997). Of these, only the three smallest variants, VEGF_165_, VEGF_145_, and VEGF_121_, are secreted as soluble factors. Cell-surface-associated heparin-like molecules enhance the binding of VEGF to its receptors. Of the three soluble variants, VEGF_165_ and VEGF_145_ contain such heparin-binding domains whereas VEGF_121_ does not.

VEGFRs are highly expressed in areas of neoangiogenesis, such as in the endothelial cells of tumour vasculature, a restricted distribution that may be exploited to provide targeted therapy, using VEGF as a delivery molecule. Fusion proteins between VEGF and diphtheria toxin are indeed toxic to endothelial cells and to Kaposi's sarcoma cell lines, and inhibit angiogenesis, in a VEGFR-dependent manner (Arora *et al*, 1999). However, the disadvantage of such systems is that each protein molecule can affect only one cell. An alternative to diphtheria toxin is the use of an enzyme that can convert large numbers of molecules of a low molecular weight noncytotoxic prodrug to a cytotoxic drug. We have tested whether the bacterial enzyme carboxypeptidase G2 (CPG2, glutamate carboxypeptidase, E.C.3.4.17.11) can be fused to VEGF, to deliver a prodrug-activating enzyme to proliferating endothelial cells.

CPG2 hydrolyses several aromatic N-substituted glutamates to release benzoic acid, phenol, and aniline mustards (Springer, 1993; Springer *et al*, 1995); or when employing a self-immolative mechanism, can release other conventional therapeutic agents, such as anthracycline antibiotics (Niculescu-Duvaz *et al*, 1999). In this study, we used the noncytotoxic prodrug CMDA that releases L-glutamic acid and benzoic acid nitrogen mustard, a potent DNA-alkylating agent (Figure 1

**Figure 1:**
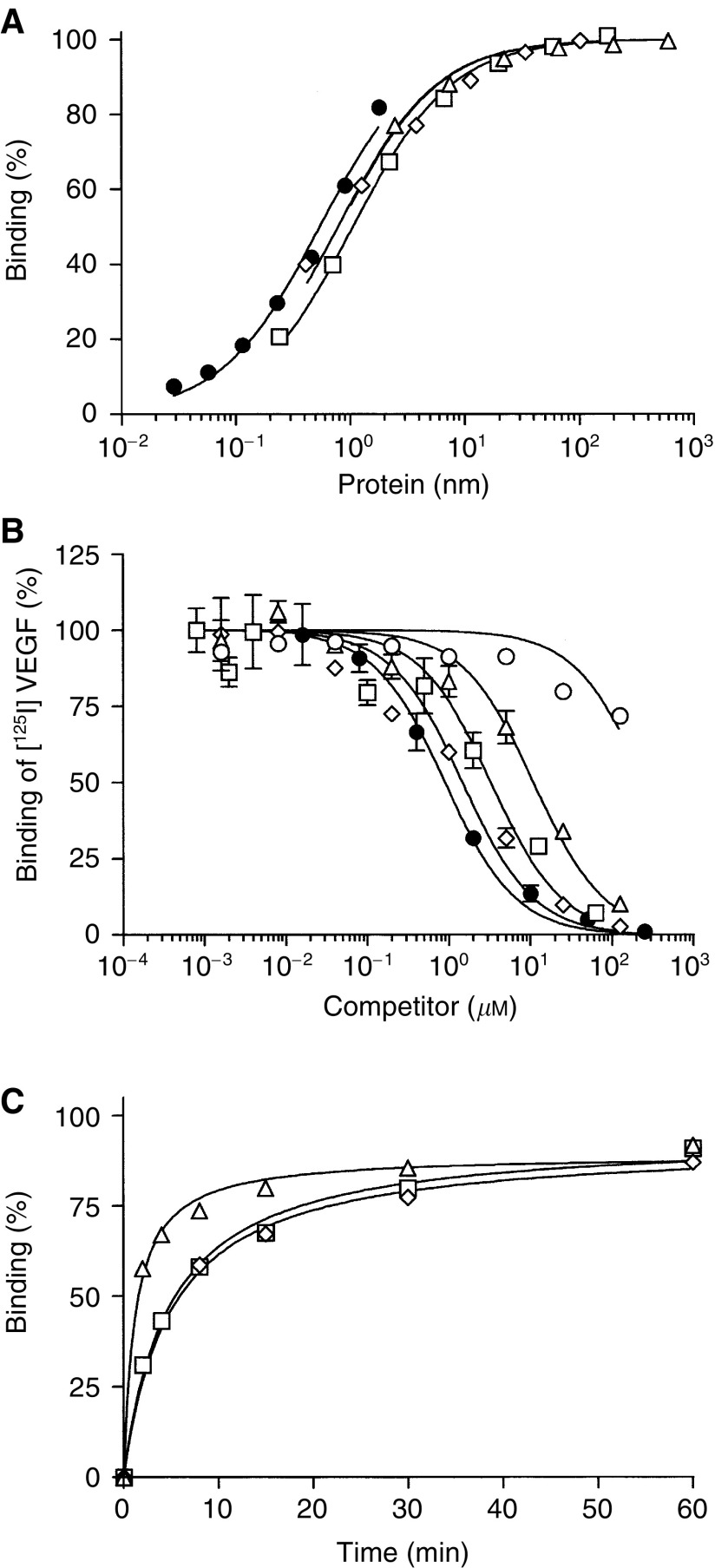
Activation of CMDA by CPG2. CPG2 cleaves the benzoic acid mustard prodrug 4-([2-chloroethyl][2-mesyloxyethyl]amino)benzoyl-L-glutamic acid (CMDA) to release L-glutamic acid and the mustard drug 4-([2-chloroethyl][2-mesoxyethyl]amino)benzoic acid, a potent DNA-alkylating agent.

). An advantage of alkylating agents is that they are cytotoxic to both cycling and noncycling cells (Frei *et al*, 1988; Teicher and Frei, 1988), such as would be found in tumours. CPG2 has previously been used in antibody-directed enzyme-prodrug therapy (ADEPT) in which it is targeted to tumours with tumour-specific antibodies (Bagshawe, 1990), an approach in clinical trial (Bagshawe *et al*, 1991; Napier *et al*, 2000). Similarly, expression of CPG2 tethered to the cell surface in gene-directed enzyme-prodrug therapy (GDEPT) leads to extracellular prodrug activation (Marais *et al*, 1997), which in preclinical studies has been shown to generate a bystander effect, where neighbouring cells that do not express CPG2 are also killed (Stribbling *et al*, 2000). By analogy with ADEPT and GDEPT, VEGF-directed delivery of CPG2 will activate CMDA external to the cells and should also lead to killing of both target and bystander cells. The targeting of CPG2 to a tumour using VEGF will also cause destruction of the tumour vasculature and thus lead to significant levels of collateral damage with consequent ischaemic death of the tumour.

In this paper, we describe the construction, expression, and characterisation of a number of fusion proteins between VEGF and CPG2 that can bind to recombinant VEGFR2, and can differentiate between human umbilical vein endothelial cells (HU-V-EC) that express VEGFR2 and human ovarian adenocarcinoma cells (SK-OV-3) that do not. Once bound, these proteins can convert CMDA to its toxic drug derivative and thereby kill the targeted cells. Further, we identify one construct, VEGF_115_-CPG2(Q)3-H_6_, with a combination of features that recommend it as a lead molecule for this targeted therapy, which we call Ligand-Directed Enzyme Prodrug Therapy or LiDEPT.

## MATERIALS AND METHODS

### Construction of plasmids encoding fusion proteins

All fusion genes were generated by PCR (Ho *et al*., 1989) and their sequences verified by automated DNA sequencing. For mammalian expression, constructs were cloned into the vector pEFPlink.2, which uses the elongation factor 1*α* (EF1*α*) promoter to direct expression in mammalian cells (19). For stable expression, the vector pMCEF− was used, which also uses the EF1*α* promoter to direct expression, but additionally incorporates a neo^R^ gene for clone selection (Marais *et al*, 1997).

### Expression and purification of fusion proteins

Mammalian cells were transfected with expression plasmids using LipofectAmine, as previously described (Marais *et al*., 1997). Expression in insect cell vectors was achieved using the BaculoGold system (Pharmingen) as previously described (Marais *et al*, 1997). For small-scale protein extraction, detergent-soluble extracts were prepared as previously described (Marais *et al*, 1996), except that *β*-mercaptoethanol was omitted from all the buffers. For bulk protein production, 10^8^ Sf9 insect cells were infected with fusion protein-encoding virus in a total of 100 ml growth medium. The cells were grown in suspension for up to 9 days. Samples of growth medium (100 *μ*l) were monitored daily for CPG2 enzyme activity. When maximum yield was reached, the conditioned growth medium was filtered (0.2 *μ*m) and dialysed against 3 × 5l 50 mM Tris-HCl pH 8, 500 mM NaCl (column loading buffer), and H_6_-tagged proteins were purified by nickel-agarose chromatography, using standard protocols. The proteins were eluted with loading buffer containing 90 mM imidazole, and dialysed extensively against 100 mM Tris-HCl pH 7.3, 260 *μ*M ZnCl_2_. The sample was adjusted to 10% final glycerol and stored at −70°C. CPG2 enzyme assays were performed using a standard assay. Briefly, samples containing CPG2 (100 *μ*l) were incubated in 900 *μ*l 100 mM Tris-HCl pH 7.3, 260 *μ*M ZnCl_2_ containing methotrexate (MTX, 55 *μ*M), and activity was determined from the rate at which the absorbance at 320 nm declined. Immunoprecipitations were performed using standard approaches, using either a CPG2 polyclonal antibody (Hogg *et al*, 1987) or the 9E10 monoclonal antibody, which binds to the myc-epitope tag (Evan *et al*, 1985).

### Receptor binding studies and affinity measurements

The apparent affinities of the fusion proteins for VEGFR2 were estimated by adapting a method of solid-phase immunoassay (Hogg *et al*, 1987). To measure the affinity of VEGF for the ligand-binding domain of VEGFR2, the binding to a construct in which the kinase domain was deleted (VEGFR2(ΔKD)m) was determined. Microtitre plates (96 well) were precoated with the 9E10 antibody (20 *μ*g ml^−1^) and nonspecific sites were blocked with 3% skimmed milk powder in phosphate buffered saline (PBS) pH 7.4 containing 0.1% Tween-20 (PBS-T). Detergent extracts containing VEGFR2(ΔKD)m were incubated overnight (4°C) and the samples were incubated with [^125^I]VEGF (Amersham Biosciences, UK), followed by three washes with PBS-T and the amount of VEGF bound was determined by scintillation counting. The binding of the fusion proteins to VEGFR2(ΔKD)m was determined as above, but using an ELISA format. Following binding of VEGFR2(ΔKD)m to the antibody-coated microtitre plates, the samples containing the various fusion proteins were incubated with the complexes and the amount of fusion protein bound was measured by ELISA using a rabbit antiserum to CPG2 followed by anti-rabbit horseradish peroxidase and developed using the tetramethylbenzidine liquid substrate system (Sigma-Aldrich, UK).

Binding affinities were also estimated using a competitive displacement radiolabel receptor assay as previously described (Keyt *et al*, 1996) but substituting VEGFR2(ΔKD)m for the KDR-IgG fusion protein, and using the fusion proteins secreted from insect cells. The apparent dissociation constant (*K*_d_) for each fusion protein was estimated from the concentration required for 50% inhibition of binding of [^125^I]VEGF to immobilised VEGFR2(ΔKD)m.

### Synthesis of CMDA

CMDA prodrug was synthesised as described previously (Springer, 1993). It was prepared as a 100 mM solution in DMSO immediately prior to use.

### Cytotoxicity studies

Human umbilical vein endothelial cells (HU-V-EC) were obtained from the American Tissue Culture Collection (ATCC) and were maintained in CS-C medium (Sigma-Aldrich, Missouri, USA), supplemented with 10% fetal calf serum and endothelial cell growth factor (Sigma-Aldrich, UK) according to the manufacturer's recommendations. Human ovarian adenocarcinoma SK-OV-3 cells were maintained in Dulbecco's modified Eagle's medium supplemented with 10% fetal calf serum.

To determine their inherent sensitivity to CMDA, HU-V-EC and SK-OV-3 cells were seeded (3 × 10^5^ cells well^−1^) in six-well tissue culture plates. After 2-day growth, the cells were incubated for 19 h with 8000, 4000, 2000, 1000, 500, 250, or 125 *μ*M CMDA that had been diluted in appropriate growth medium. A proportion of the cells (approximately 30% for HU-V-EC or approximately 3% for SK-OV-3) were replated and allowed to grow for 5 days, when the surviving fraction was determined from the incorporation of [^3^H]thymidine (0.4 *μ*Ci ml^−1^, 6 h).

For LiDEPT-dependent killing of the different cell lines, confluent monolayers of cells were exposed to varying concentrations of fusion proteins in complete growth medium (37°C, 30 min), followed by three washes with growth medium to remove unbound protein. The cells were then incubated in growth medium containing different concentrations of CMDA (19 h) and cell survival determined as described above.

### *In vivo* stability studies

All experiments were conducted in accordance with the United Kingdom Co-ordinating Committee on Cancer Research guidelines (Workman *et al*., 1998). A group of 18 BALB/C nude mice was injected i.p. with 50 units of VEGF_115_CPG2(Q)3-H_6_ in 200 *μ*l PBS. At intervals of 4, 8, 12, 16, 20, 24 h after injection, triplicate blood samples were collected, and the CPG2 enzymic activity in the samples determined by assay against the model substrate methotrexate as previously described (Stribbling *et al*, 1997)

## RESULTS

### Construction of fusion proteins between VEGF and CPG2

**Figure 2:**
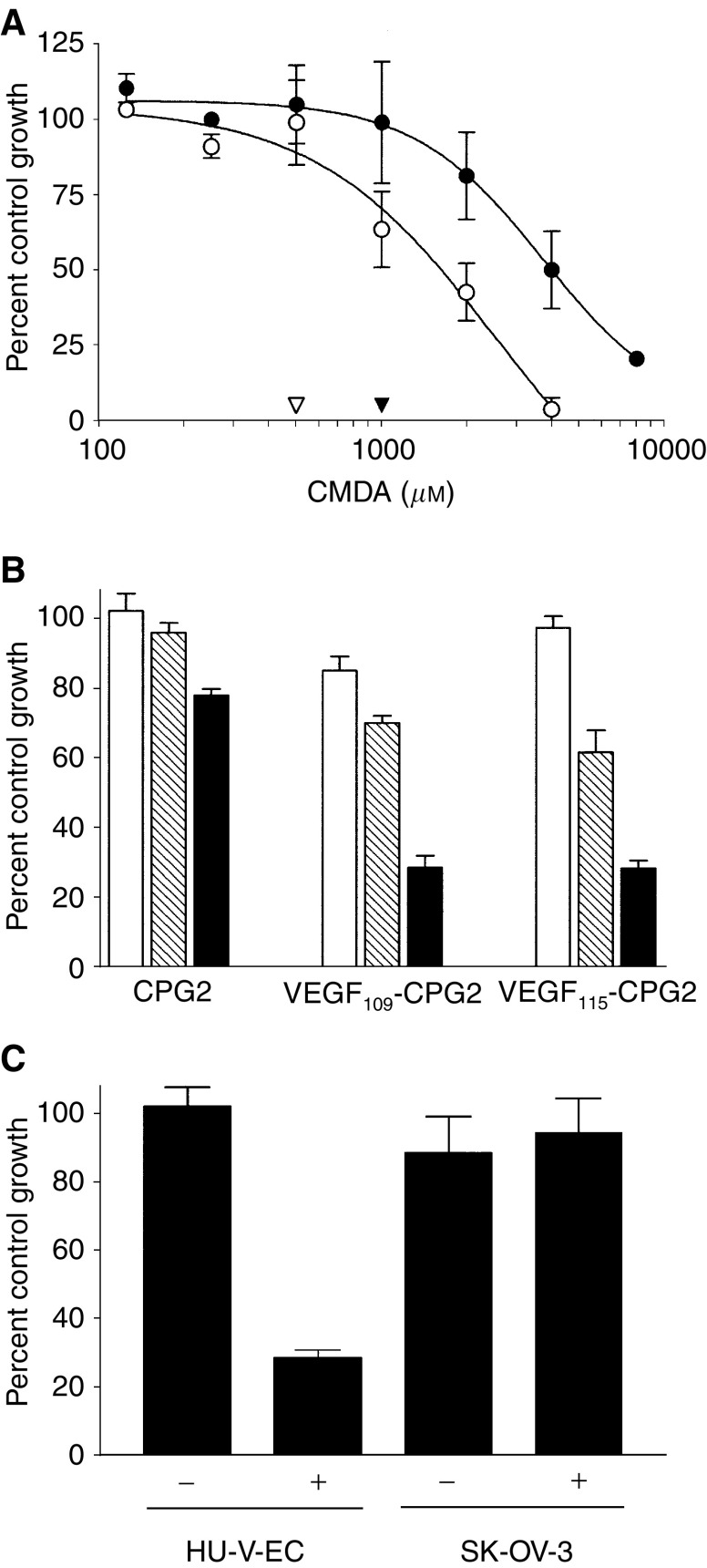
Schematic representations of the structures of CPG2(Q)3-H_6_ and fusion proteins. SP_erb_ and SP_v_: signal peptides of c-erb B2 and VEGF, respectively. CPG2 23-415: amino acids 23–415 of CPG2, showing the relative positions of the three asparagine to glutamine mutations (Q) required to avoid glycosylation and concomitant loss of CPG2 activity. H_6_: oligohistidine tag for purification by nickel-agarose chromatography. VEGF_165_, VEGF_109_, VEGF_115_, and VEGF_161_: amino acids 1–165, 109, 115, and 161, respectively, of VEGF_165_. Black box: the relative position of the heparin-binding sequences of the longer forms of VEGF.

Since CPG2 is a highly versatile protein, two classes of fusion protein between VEGF and CPG2 were considered. VEGF was fused either to the N-terminus or to the C-terminus of CPG2. For these studies, the aim was for these proteins to be secreted, so that the structurally important disulphide bonds of VEGF could form with high efficiency in the oxidising environment of the endoplasmic reticulum, and for glycosylation of VEGF to occur. Therefore, all constructs were provided with signal peptides. For those fusions in which VEGF was fused to the N-terminus of CPG2, the VEGF signal peptide was employed. For those fusion proteins that had CPG2 in the N-terminal position, the c-erbB2 signal peptide was employed, as this approach was previously shown to direct CPG2 to mammalian secretory pathways (Marais *et al*, 1997). However, since CPG2 becomes glycosylated and consequently is inactivated in this pathway, a version of CPG2 (CPG2(Q)3) was used, in which all three glycosylation sites were mutated to prevent this event. The VEGF and CPG2(Q)3 were separated by either a nine amino-acid linker (EF(G)_5_TA) or a minimal linker (GS). A schematic diagram of the various constructs is shown in Figure 2

, and a description of their amino-acid structures is provided in Table 1

**Table 1:** Structures of the fusion proteins

**Protein**	**Predicted primary sequence**	***M*_r_ (e)**	***M*_r_ (o)**
CPG2(Q)3-H_6_	(c-erb B2 aa 1-27)-GS-(CPG2(Q)3 aa 23-415)-EF(H)_6_AS	43 731	45 000
CPG2(Q)3-VEGF_165_	(c-erb B2 aa 1-27)-GS-(CPG2(Q)3 aa 23-415)-EF(G)_5_TA-(VEGF aa 1-165)	62 272	59–61 000
CPG2(Q)3-VEGF_161_-H_6_	(c-erb B2 aa 1-27)-GS-(CPG2(Q)3 aa 23-415)-EF(G)_5_TA-(VEGF aa 1-161)-EF(H)_6_AS	63 215	60–62 000
CPG2(Q)3-VEGF_109_	(c-erb B2 aa 1-27)-GS-(CPG2(Q)3 aa 23-415)-EF(G)_5_TA-(VEGF aa 1-109)	55 657	53–55 000
CPG2(Q)3-VEGF_109_-H_6_	(c-erb B2 aa 1-27)-GS-(CPG2(Q)3 aa 23-415)-EF(G)_5_TA-(VEGF aa 1-109)-EF(H)_6_AS	57 135	54–59 000
VEGF_161_-CPG2(Q)3-H_6_	(VEGF aa -26-161)-GS-(CPG2(Q)3 aa 23-415)-EF(H)_6_AS	61 710	61–63 000
VEGF_115_-CPG2(Q)3-H_6_	(VEGF aa -26-115)-GS-(CPG2(Q)3 aa 23-415)-EF(H)_6_AS	56 386	55–61 000

Sequences of linkers between VEGF and CPG2-derived moieties and of oligohistidine tags for purification by nickel-agarose chromatography are given in the one-letter amino-acid code (22); *M*_r_ (e), expected relative subunit molecular weight; *M*_r_ (o), observed subunit molecular weight; aa, amino acids. c-erb B2 aa 1–27 encode the c-erb B2 signal peptide. VEGF aa -26-1 encode the VEGF signal sequence.

. The final four C-terminal amino acids of VEGF_165_ were deleted in VEGF_161_-CPG2(Q)3-H_6_ since they contain three positively charged residues, which, if incorporated between fused VEGF and CPG2(Q)3 moieties, might be subject to recognition and cleavage by serine proteases such as trypsin. VEGF_109_ arose accidentally as a PCR-generated truncation of VEGF_165_, and was retained for study. VEGF_115_ was generated as a refinement of VEGF_109_, being truncated exactly at the junction of the heparin-binding domain. Finally, to facilitate purification, polyhistidine tags (H_6_) were fused to the C-termini of a number of the constructs to allow purification by nickel-agarose chromatography. The observed relative masses of the constructs were determined from their electrophoretic mobilities (Table 1). In each case, the relative mass was similar to the value that would be predicted from the primary sequence.

### Expression and characterisation of VEGF-CPG2 fusion proteins in mammalian cells

**Figure 3:**
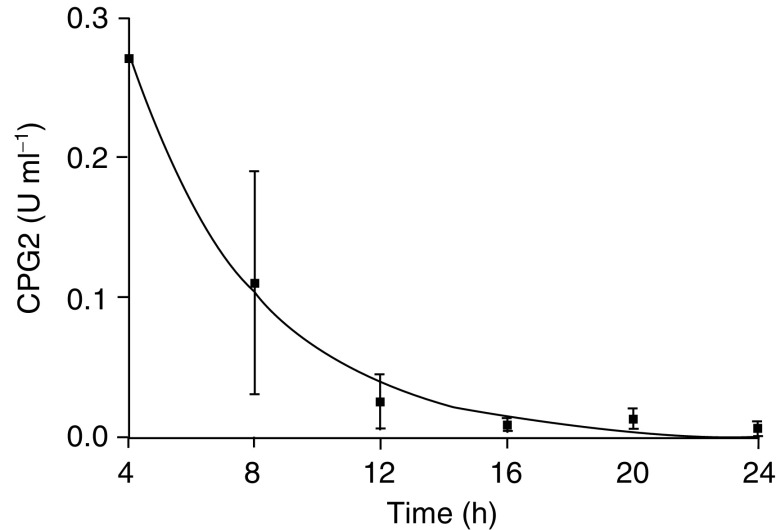
Expression of VEGF-CPG2 fusion proteins in mammalian cells. (**A**) CPG2 enzyme activity assays. COS-7 cells were transiently transfected with expression constructs for CPG2(Q)3-VEGF_165_ (▴,▵), CPG(Q)3-VEGF_109_ (♦,⋄), CPG2(Q)3-H_6_ (•,○) and *β*-galactosidase (▪, □). CPG2 enzyme assays were performed on ∼1/30th of the conditioned growth medium (100 *μ*l; ▵,⋄,□,○) or detergent cell extracts (∼10 *μ*g of protein; ▴,♦,▪,•). Data are means of three separate transfections, except for *β*-galactosidase expression, which are mean data from two separate transfections. (**B**) Western blotting of fusion proteins. Proteins from the conditioned medium from COS cells expressing *β*-galactosidase (*β*-gal) (lane 1), CPG2(Q)3-H_6_ (lane 2), CPG2(Q)3-VEGF_165_ (lane 3), and CPG(Q)3-VEGF_109_ (lane 4) were immunoprecipitated using a polyclonal antibody to CPG2 and subjected to Western blotting for fusion protein expression. The positions of migration of size markers (× 10^−3^) are shown to the right and arrowheads to the left identify the proteins as follows: upper, CPG2(Q)3-VEGF_165_, middle CPG2(Q)3-VEGF_109_, and lower, CPG2(Q)3-H_6_. The figure is a composite of two separate segments of one gel. (**C**) Heparin binding of VEGF-CPG2 fusion proteins. Conditioned medium from COS-7 cells transiently expressing *β*-gal (lane 1), CPG2(Q)3-H_6_ (lane 2), CPG2(Q)3-VEGF_165_ (lane 3), and CPG2(Q)3-VEGF_109_ (lane 4) was analysed for the ability of the fusion proteins to bind to heparin-Sepharose. Western blotting revealed the bound proteins and the positions of migration of size markers (× 10^−3^) are shown to the left. The arrowhead to the right points towards the position of migration of CPG2(Q)3-VEGF_165_. The figure is a composite of two separate segments of one gel.

As a first step, CPG2(Q)3-VEGF_165_, CPG2(Q)3-VEGF_109_, and CPG2(Q)3-H_6_ were transiently expressed (see Table 1, Figure 2) in COS-7 cells. The filtered, conditioned growth media from the cells was tested for CPG2 activity, by examining its ability to hydrolyse the CPG2 substrate methotrexate (MTX). As expected, CPG2 activity was detected in the conditioned growth medium from CPG2(Q)3-VEGF_165_, CPG2(Q)3-VEGF_109_, and CPG2(Q)3-H_6_ expressing cells, but not from control cells expressing *β*-galactosidase (*β*-gal)(Figure 3A

). There was also detectable CPG2 activity in detergent extracts from these cells (Figure 3A). In Western blotting of the conditioned medium, CPG2 fusion proteins were not detected (data not shown). However, fusion proteins of the expected sizes (see Table 1) were detected in immunoprecipitates of the conditioned medium that used a CPG2 specific antibody to capture the secreted CPG2-fusion proteins (Figure 3B, lanes 2–4). The VEGF moieties of the fusion proteins were then examined for binding to heparin. Conditioned medium from the cells was incubated with heparin-Sepharose, and the heparin-bound proteins were analysed by Western blotting. As expected, only CPG2(Q)3-VEGF_165_ bound to heparin-Sepharose (Figure 3C, lane 3), because CPG2(Q)3-VEGF_109_ does not contain the heparin-binding domain (Figure 3c, lane 4). *β*-gal and CPG2(Q)3-H_6_ also failed to bind to heparin (Figure 3C, lanes 1,2).

Next, the expression of the additional fusion proteins was tested. VEGF_161_-CPG2(Q)3-H_6_ and VEGF_115_-CPG2(Q)3-H_6_ are also secreted and accumulate in growth medium to higher levels than the fusions with the N-terminal CPG2(Q)3 moiety (Table 2

**Table 2:** Expression of fusion proteins between CPG2(Q)3 and VEGF in COS-7 cells

	**Total CPG2 activity (U well^−1^) in:**		**Relative CPG2-specific**	**Relative heparin-binding**
**Protein**	**Cells**	**Medium**	**activity (%)**	**activity (%)**
CPG2(Q)3-VEGF_165_	0.0023 (±0.0005)	0.021 (±0.001)	110 (±11)	100
CPG2(Q)3-VEGF_109_	0.0077 (±0.0005)	0.023 (±0.001)	143 (±13)	2.1
VEGF_161_-CPG2(Q)3-H_6_	0.0098 (±0.0013)	0.037 (±0.009)	102 (±13)	99
VEGF_115_-CPG2(Q)3-H_6_	0.0009 (±0.0001)	0.066 (±0.011)	140 (±19)	10.2
CPG2(Q)3-H_6_	0.084 (±0.10)	0.139 (±0.004)	100	2.4

The CPG2 in conditioned media and cell extracts was measured using quantitative Western blotting to estimate the relative protein levels by phosphorimaging. The relative activity of the different fusion proteins could then be estimated using the standard enzyme assay. Quantitative Western blotting was also used to determine the relative efficiency of binding of the fusion proteins to heparin-Sepharose. Data shown for measurements of total activity and relative activity are means (±s.d.) of three transfections.

). In all cases, the secreted product accumulates to greater amounts in the medium than in the cell extracts (Table 2). VEGF_115_-CPG2(Q)3-H_6_ is secreted particularly efficiently, with over 98% of the total expressed activity found in the growth medium. This protein is also the most highly expressed of all the CPG2-VEGF fusion proteins tested. To estimate the specific activity of the CPG2 moiety of the fusion proteins, the concentrations of CPG2 protein in the conditioned media were measured using quantitative Western blotting. The specific activities of the different fusion proteins could then be estimated using the standard enzyme assay. All of the fusion proteins had levels of enzyme activity equivalent to, or higher than the nonfused secreted form of CPG2(Q)3 (CPG2(Q)3-H_6_; Table 2). Indeed, the fusions with the shorter forms of VEGF both had greater activity (approximately 140%) than secreted CPG2(Q)3. However, there does not appear to be any difference in CPG2 activity when the relative orientation of the VEGF- and CPG2-derived moieties is compared. Quantitative Western blotting was also used to determine the relative efficiency of binding of the fusion proteins to heparin-Sepharose as in Table 2. CPG2(Q)3-VEGF_165_ and VEGF_161_-CPG2(Q)3-H_6_ both bound to heparin with similar efficiencies, whereas the binding of the fusions with the shorter forms of VEGF was severely reduced (Table 2).

### Expression of VEGF-CPG2 fusion proteins in Sf9 cells

The above data establish that CPG2-VEGF fusion proteins can be produced that retain CPG2 enzyme activity and heparin binding in the VEGF moiety. These fusion proteins were tested for binding to the VEGF receptor, VEGFR2, to ascertain whether they could mediate killing of mammalian cells. To perform these experiments, large amounts of fusion protein were expressed in Sf9 insect cells.

Baculoviruses encoding CPG2(Q)3-H_6_, VEGF_115_-CPG2(Q)3-H_6_, CPG2(Q)3-VEGF_109_-H_6_, and VEGF_161_-CPG2(Q)3-H_6_, were generated and used to infect Sf9 insect cells. The fusion proteins were purified from conditioned growth media by single-step nickel-agarose affinity chromatography, and were judged to be 99% pure by silver staining (data not shown) except for VEGF_161_-CPG2(Q)3-H_6_, which required an extra heparin affinity chromatography step to reach a similar degree of purity (data not shown).

**Figure 4:**
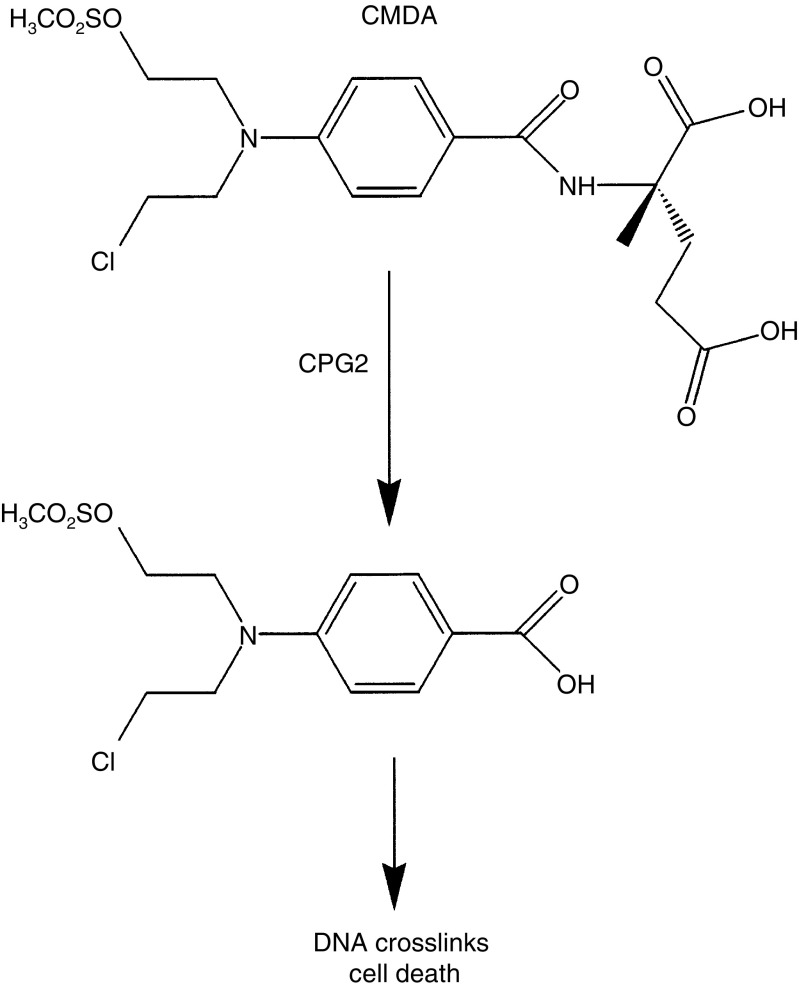
VEGF-CPG2(Q)3 and CPG2(Q)3-VEGF fusion proteins are disulphide-linked dimers. Affinity-purified CPG2(Q)3-H_6_, VEGF_115_-CPG2(Q)3-H_6_, CPG2(Q)3-VEGF_109_-H_6_, and VEGF_161_-CPG2(Q)3-H_6_ proteins were heated in either reducing (lanes 1–4) or nonreducing (lanes 5–8) conditions, subjected to polyacrylamide gel electrophoresis and were identified after immunoblotting using a rabbit anti-CPG2 serum and ^125^I-Protein A. Closed arrowheads show the positions of migration of monomers and open arrowheads show the positions of migration of dimers. The positions of migration of size markers (× 10^−3^) are shown to the right. Asterisks mark the positions of migration of a breakdown product derived from VEGF_161_-CPG2(Q)3-H_6_ protein.

CPG2 is a homodimeric protein that is denatured by heat treatment in the presence of SDS, and so migrates as monomers in polyacrylamide gels following heat treatment (Spooner *et al*, 2000). VEGF is also a homodimer, but it is stabilised by disulphide linkage. To assess whether the VEGF moieties were stabilised by disulphide linkages, the different fusion proteins were subjected to heat treatment under reducing or nonreducing conditions. As expected, CPG2(Q)3-H_6_ migrated as a monomer under both reducing and nonreducing conditions (Figure 4

, lanes 1, 5). VEGF_115_-CPG2(Q)3-H_6_, CPG2(Q)3-VEGF_109_-H_6_, and VEGF_161_-CPG2(Q)3-H_6_ also migrated as monomers under reducing conditions (Figure 4, lanes 2–4), but under nonreducing conditions migrated with sizes that were consistent with dimerization (Figure 4, lanes 6–8). A contaminating protein, presumably a degradation product related to CPG2, is routinely found in preparations of VEGF_161_-CPG2(Q)3-H_6_ (Figure 4, lanes 4, 8). We conclude that the VEGF moieties in these fusion proteins are stabilised by disulphide linkages. These fusion proteins were then tested for the ability to bind to the ligand-binding domain of VEGFR2. For these studies, the extracellular and transmembrane domains of VEGFR2 were expressed as a 9E10-tagged protein in insect cells. This construct, which has the kinase domain (KD) of VEGFR2 deleted, included codons −20–809 of VEGFR2 fused to a 9E10 monoclonal antibody epitope (VEGFR2(ΔKD)m).

**Figure 5:**
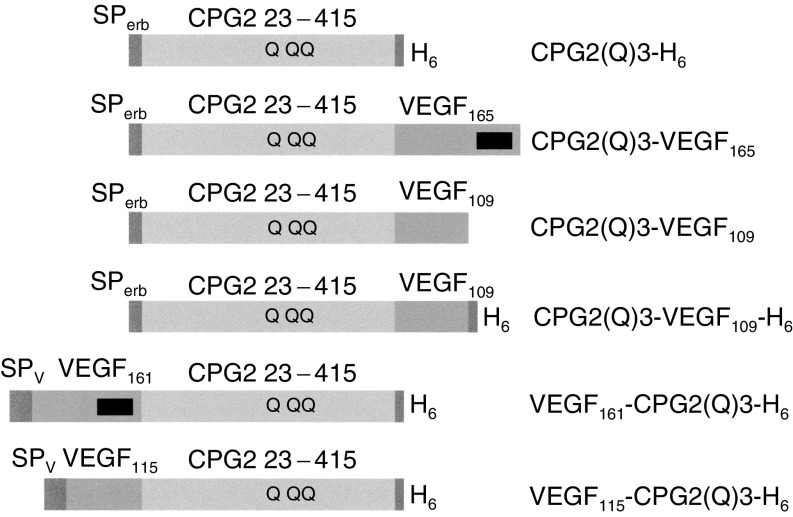
*In vitro* binding assays: (**A**) ELISA showing binding of [^125^I]VEGF (•), VEGF_115_-CPG2(Q)3-H_6_ (▵), VEGF_161_-CPG2(Q)3-H_6_ (□),or CPG2(Q)3-VEGF_109_-H_6_ (⋄) to VEGFR2(ΔKD)m. (**B**) RIA showing competition of CPG2(Q)3-H_6_ (○), VEGF_115_-CPG2(Q)3-H_6_ (▵), VEGF_161_-CPG2(Q)3-H_6_ (□), CPG2(Q)3-VEGF_109_-H_6_ (⋄), or VEGF_165_ (•) with [^125^I]VEGF for VEGFR2(ΔKD)m. (**C**) Kinetics of binding of VEGF_115_-CPG2(Q)3-H_6_ (▵),VEGF_161_-CPG2(Q)3-H_6_ (□),or CPG2(Q)3-VEGF_109_-H_6_ (⋄) to VEGFR2(ΔKD)m captured by immobilised 9E10 monoclonal antibody, measured by ELISA.

For VEGF binding studies, VEGFR2(ΔKD)m in a 96-well plate ELISA format was used. The plates were seeded with the 9E10 monoclonal antibody and VEGFR2(ΔKD)m was captured on the immobilised 9E10 antibody. [^125^I]VEGF bound to this immobilised VEGFR2(ΔKD)m with high affinity (*K*_d_∼0.5 nM). The fusion proteins were incubated with the captured VEGFR2(ΔKD)m and binding was determined. All of the fusion proteins bound with similar affinities, with *K*_d_ values of approximately 0.5–1.1 nM (Figure 5A

, Table 3

**Table 3:** Binding characteristics of fusion proteins between CPG2(Q)3 and VEGF expressed in insect cells

**Protein**	**ELISA *K*_d_ (nM)**	**Competition RIA *K*_d_ (nM)**	**50% binding *t*_50_ (s)**
CPG2(Q)3-VEGF_109_-H_6_	0.75 (±0.05)	1.3 (±0.1)	332
VEGF_161_-CPG2(Q)3-H_6_	1.06 (±0.66)	3.3 (±0.3)	330
VEGF_115_-CPG2(Q)3-H_6_	0.51 (±0.08)	9.6 (±0.6)	77
CPG2(Q)3-H_6_	ND	No competition	ND
[^125^I]-VEGF	0.51 (±0.06)	ND	ND

VEGFR2(ΔKD)m was captured on the 9E10 antibody immobilised in 96-well plates. The fusion proteins were incubated with the captured VEGFR2(ΔKD)m and binding was determined. We also used a competition RIA where binding of [^125^I]VEGF to VEGFR2(ΔKD)m occurs in the presence of competing fusion proteins. The rate of binding was also determined, by exposing the fusion proteins to immobilised VEGFR2(ΔKD)m for short times before washing out the protein and measuring how much fusion protein was bound.

).

A competition RIA was used where binding of [^125^I]VEGF to VEGFR2(ΔKD)m occurs in the presence of competing fusion proteins. Using this analysis, the binding of the fusion proteins was estimated to be slightly higher, with *K*_d_ values from 1.3–9.6 nM (Figure 5B, Table 3). CPG2(Q)3-H_6_ protein did not compete for [^125^I]VEGF binding in this assay (Table 3). Finally, the rate of binding was measured, by exposing the fusion proteins to immobilised VEGFR2(ΔKD)m for short times before washing out the protein and measuring how much fusion protein was bound. VEGF_115_CPG2(Q)3-H_6_ reaching half-maximal binding in 77 s (Figure 5C, Table 3). The binding of CPG2(Q)3VEGF_109_-H_6_ and VEGF_161_CPG2(Q)3-H_6_ was somewhat slower, reaching half-maximal binding after about 330 s.

### Kinetic analysis of VEGF_115_CPG2(Q)3-H_6_

Taken together, the above data suggest that VEGF_115_CPG2(Q)3-H_6_ is the most suitable candidate for the LiDEPT approach. It displays rapid binding to receptor, is secreted efficiently, is the most highly expressed, and has the highest specific activity. We therefore subjected this protein to detailed kinetic analysis for the CPG2 activity. CPG2 produced in bacteria has a *K*_m_ for MTX of 8 *μ*M (Sherwood *et al*, 1985). Similarly, CPG2 expressed in the cytosol of mammalian and insect cells also have *K*_m_ values for MTX of approximately 7 *μ*M (Napier *et al*, 2000). Secreted CPG2(Q)3-H_6_, has a *K*_m_ of 29.5 *μ*M MTX and VEGF_115_-CPG2(Q)3-H_6_ a *K*_m_ of 37.5 *μ*M MTX (mean of two independent preparations). Again, a slight increase in catalytic activity was noted for this fusion protein (140%, Table 2). Thus, the fusion of VEGF domains to CPG2(Q)3 does not appear to affect the affinity for substrate as compared to secreted CPG2(Q)3, and can result in a moderate increase in catalytic activity.

### Fusion proteins of CPG2 and VEGF can direct cytotoxicity to VEGFR2-positive cells

**Figure 6:**
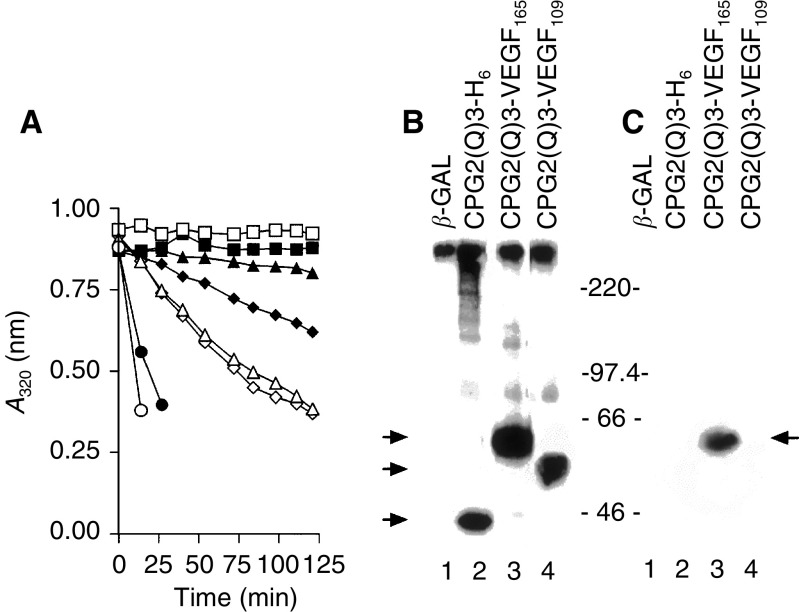
Cytotoxicity studies. (**A**) Sensitivity of HU-V-EC (○) and SK-OV-3 cells (•) to treatment with the prodrug CMDA. Arrowheads show the maximum concentrations of CMDA that the HU-V-EC (▿) and SK-OV-3 cells (▾) cells can tolerate without measurable cell death. (**B**) Effect on the survival of HU-V-EC cells of preincubation with CPG2(Q)3-H_6_, CPG2(Q)3-VEGF109-H_6_, or VEGF115CPG2(Q)3-H_6_, at doses of 0.5 nm (plain bars), 5 nm (shaded bars), or 50 nm (filled bars) and subsequent treatment with 500 *μ*M CMDA. (**C**) Sensitivity of HU-V-EC and SK-OV-3 cells to CMDA challenge (500 and 1000 *μ*M, respectively) after a 30 min incubation with 50 nm VEGF_115_CPG2(Q)3-H_6_.

VEGF-CPG2 fusion proteins were next assessed for their ability to generate a targeted increase in the cytotoxicity of CMDA towards HU-V-EC cells, which express receptors for VEGF. Initially, the sensitivity of HU-V-EC cells to CMDA alone was established. The IC_50_ of CMDA in HU-V-EC cells was 1587 (±) 224 *μ*M (Figure 6A

). The highest dose of CMDA that produced no cytotoxicity in HU-V-EC cells was 500 *μ*M (open arrowhead, Figure 6A).

Confluent HU-V-EC cells were incubated with three doses of VEGF_115_-CPG2(Q)3-H_6_, CPG2(Q)3-VEGF_109_-H_6_, or CPG2(Q)3-H_6_ (50, 5, or 0.5 nM) in complete growth medium for 30 min, after which the unbound proteins were removed by rinsing × 3. These cells were then incubated in the previously established nontoxic dose of CMDA (500 *μ*M), and cell survival then determined in our standard assay. Only a slight increase in cytotoxicity was seen with increasing concentrations of CPG2(Q)3-H_6_ (Figure 6B). By contrast, a marked protein-dose-dependant reduction in survival was seen following exposure to VEGF_115_-CPG2(Q)3-H_6_, and VEGF_109_-CPG2(Q)3-H_6_, indicative of these proteins having resisted rinsing, being bound, presumably to the VEGFRs of the cells. To confirm that this increased cytotoxicity is indeed the result of binding to VEGFRs, a similar experiment was performed, comparing HU-V-EC cells to SK-OV-3 cells, which by contrast do not express VEGFRs. As previously, the sensitivity to CMDA alone was first established. The IC_50_ of CMDA in SK-OV-3 cells was 4323 (±473) *μ*M (Figure 6A) very similar to our previous estimate (Marais *et al*, 1997). The highest dose of CMDA that produced no cytotoxicity in SK-OV-3 cells was 1000 *μ*M (filled arrowhead, Figure 6A). HU-V-EC and SK-OV-3 cells were treated with 50 nM VEGF_115_-CPG2-H_6_, washed three times, and then treated with subcytotoxic doses of CMDA (500 and 1000 *μ*M, respectively). This results in an increase in cytotoxicity (75%) in HU-V-EC cells, but not in SK-OV-3 cells (Figure 6), confirming that the fusion protein is binding to VEGFRs on HU-V-EC cells.

Thus, the cytotoxic drug is being generated from concentrations of CMDA similar to those achieved in the plasma of human patients during a phase I/II ADEPT clinical trial (Martin *et al*, 1997).

### Stability of fusion protein *in vivo*

**Figure 7:**
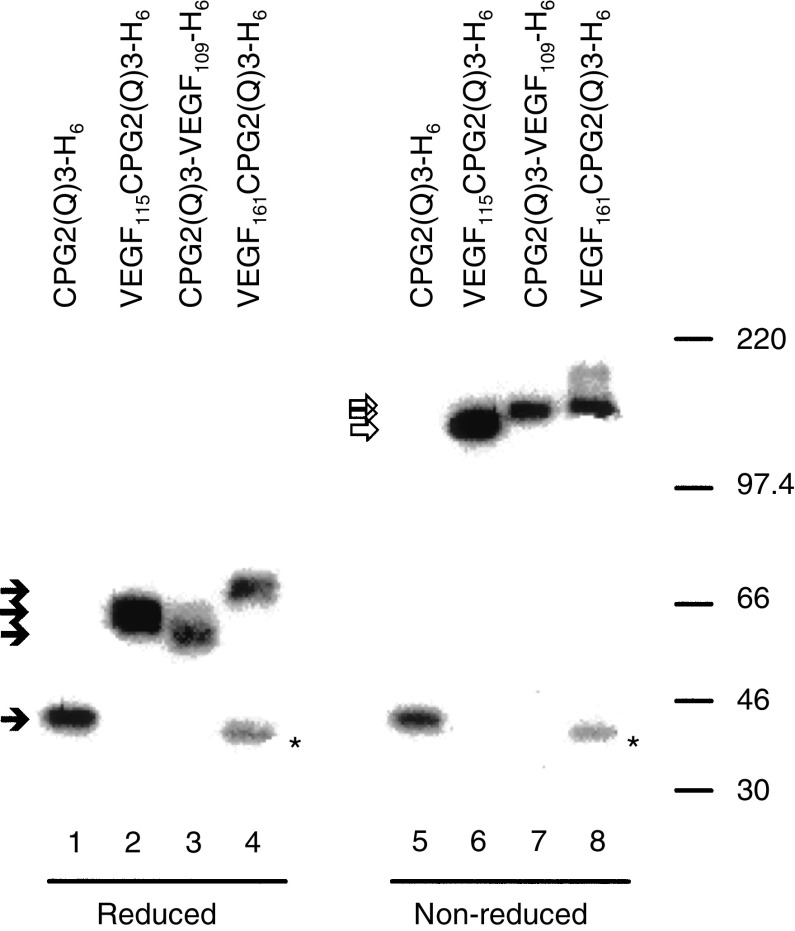
Balb/c nude mice were injected i.p. with 50 U CPG2. At intervals as indicated, blood was collected and analysed for CPG2 activity. The line is a fit to a one-phase exponential decay with a half-life of 2.8 h. Each point represents the mean of three separate determinations±s.e.m.

When VEGF_115_-CPG2-H_6_ was injected i.p. into mice, the plasma levels of enzymic CPG2 activity decayed with a one-phase exponential half-life of 3 h (Figure 7

), similar to that previously observed (4 h) with an antibody fragment-CPG2 conjugate, known as A5B7-F(ab')2-CPG2, used in ADEPT (Sharma *et al*, 1990). The presence of enzymic activity implies the presence of intact dimers, and we anticipate that this stability in the circulation will also allow localisation similar to that seen in ADEPT (Napier *et al*, 2000), a therapy now in phase I/II trial.

## DISCUSSION

The approach of targeting cytotoxicity via a fusion protein has a long and successful history. Prime examples are the fusions of cell-binding ligands with the catalytic subunit of *Pseudomonas* exotoxin A, which lacks cell-binding activity. The first example was fusion with transforming growth factor-type alpha (Chaudhary *et al*, 1987). Since then, a wide range of fusion partners have been considered, including interleukins (Chaudhary *et al*, 1989; Batra *et al*, 1991, 1992; Kreitman *et al*, 1992; Gawlak *et al*, 1993; Kunwar *et al*, 1993; Francisco *et al*, 1995; Siegall *et al*, 1995), other growth factors (Prior *et al*, 1991; Gawlak *et al*, 1993; Kunwar *et al*, 1993; Mesri *et al*, 1993; Siegall *et al*, 1995), single chain antibody fragments (Chaudhary *et al*, 1989; Batra *et al*, 1991, 1992; Kreitman *et al*, 1992; Francisco *et al*, 1995) and hormones (Ben Yehudah *et al*, 1999). Such studies have demonstrated that a wide range of ligands can be regarded as potential antitumour-targeting devices, and a wide range of cellular receptors can be viewed as targets. Furthermore, complete regressions have been recorded (Brinkmann *et al*, 1991), underlining the success of this approach.

We considered the VEGFRs as targets for an approach to deliver not a toxin, but an enzyme with catalytic activity capable of converting large numbers of molecules of a nontoxic prodrug to a toxic drug. We show that VEGF-CPG2 fusion proteins retain activities of both parental molecules, irrespective of whether they are the N- or C-terminal moiety, and that they retain these characteristics if either very short or longer linkers are used to join them. If provided with either the VEGF signal sequence or the c-erb B2 signal sequence, all the fusions are secreted into the growth medium, from where they could be purified to near homogeneity by single-step nickel-agarose chromatography. All the fusions are disulphide-linked dimers as expected. Those with shorter VEGF moieties show a moderate (1.4-fold) increase in CPG2 activity. Native CPG2 is active as a dimeric protein, while CPG2(Q)3 has a comparatively reduced activity owing to a reduction in dimer stability (Spooner *et al*, 2000). It is therefore possible that fusion with the shorter forms of VEGF compensates for the deleterious effect of the (Q)3 mutations by improving dimer stability. These fusion proteins bind VEGFR2 domains with an affinity close to that of VEGF, and one in particular, VEGF_115_-CPG2(Q)3-H_6_ binds very rapidly. It retains specificity for VEGFR2, and can differentiate between endothelial cells that express VEGFRs (HU-V-EC) and those that do not (SK-OV-3). Once delivered to cells, the proteins can convert the prodrug CMDA to its toxic metabolite, thus killing targeted cells.

Although expression of VEGFR2 is high in the neoangiogenic regions of tumour vasculature, some normal organs also express VEGFRs (Blaauwgeers *et al*, 1999; Kim *et al*, 1999). Both receptors are expressed predominantly in endothelial cells, but a few additional cell types express one or both of these receptors. Despite these caveats, recognition of VEGFRs as therapeutic targets for cancer is likely to be of value. Indeed, VEGF-diphtheria toxin fusion proteins are toxic to endothelial cells and Kaposi's sarcoma cell lines, inhibit angiogenesis and retard tumour growth, in a VEGFR-dependent manner (6). Since nearly all tumours induce local angiogenesis with associated high levels of VEGFR expression, VEGF-derived targeting molecules may have wide application for therapy.

VEGF-CPG2 fusion proteins should direct CPG2 activity to cells expressing VEGFRs, principally those activated endothelial cells in tumour vasculature. Once localised, these proteins can convert a nontoxic prodrug into a cytotoxic drug, thus providing targeted cell death of tumour neovasculature. Such a strategy might be expected to lead to cell death of targeted cells, plus a ‘bystander’ component of neighbouring nontargeted cells killed by high concentrations of soluble drug near the targeted cells, and perhaps associated with a further bystander effect via ischaemia. Since halting angiogenesis suppresses carcinoma cell invasion (Skobe *et al*, 1997), VEGFR-targeted CPG2 might also reduce spread of the disease. The features of the VEGF-CPG2 fusion proteins we describe here suggest that targeted therapy by LiDEPT is a feasible strategy for the targeting of activated endothelial cells in a tumour.

In summary, we have developed a VEGF_115_-CPG2(Q)3-H_6_ fusion protein that is highly expressed, efficiently secreted, has a high specific activity, binds rapidly to its target, can direct prodrug cytotoxicity to VEGFR2-expressing cells, and is a candidate for a new approach to cancer therapy.
